# Risky driving behaviors for road traffic accident among drivers in Mekele city, Northern Ethiopia

**DOI:** 10.1186/1756-0500-4-535

**Published:** 2011-12-13

**Authors:** Abrahim Hassen, Ameyu Godesso, Lakew Abebe, Eshetu Girma

**Affiliations:** 1 Tigray Regional Health Bureau, Mekele, Ethiopia; 2 Department of Health Education and Behavioral Sciences, Jimma University, Jimma, Ethiopia

## Abstract

**Background:**

Due to its perception as a disease of development, road traffic accident and related injuries tend to be under recognized as a major health problem in developing countries. However, majority of the world's fatalities on the roads occur in low income and middle income countries. Since the main cause of road traffic accident is attributed to human risky behaviors, it is important to identify significant factors for risky behaviors of drivers.

**Methods:**

A quantitative cross-sectional study with a sample size of 350 drivers was conducted in April 2011. The study was conducted among Taxi, Bajaj (three tire vehicles) and private owned car drivers. After proportion to size allocation for Taxi (75), Baja (103) and private owned car (172) drivers, we used systematic random sampling method to identify illegible study subjects. Data was collected with face to face interview using a pretested questioner. Univariate, bivariate and multivariate analysis was done using SPSS version 16.

**Results:**

The mean age of the respondents was 28.7 (SD 9.9). Majority were 339 (96.9%) males. Significant number of the study subjects 233 (66.6%) had risky driving behaviors. More than a quarter 100 (28.6%) had less knowledge about basic traffic signs. Majority of drivers 181 (51.7%) had negative attitude towards risky driving behaviors. Significant percent of them 148 (42.3%) had a habit of using mobile phone while driving vehicle and 28 (9.7%) had experience of driving after drinking alcohol. All the Bajaj, 97(62.6%) house car and 58(37.4%) taxi unfasten their seat belt while driving. Majority 303 (86.6%) followed the recommended speed limit of driving. About 66 (18.9%) of them had experience of punishment or warning by traffic polices in the previous 1 year and 77 (22%) ever had car accident while driving.

**Conclusions:**

Drivers of secondary education and with high average monthly income were more likely to have risky driving behavior. Having supportive attitude towards risky driving behaviors and not getting advice about risky driving from significant others increases the likelihood of developing risky driving behavior. Interventions targeted at developing negative attitude towards risky driving behaviors on drivers and significant others should be implemented to bring positive behavior change. The interventions need to be segmented with educational status and income.

## Background

Road traffic accident is a major but neglected public health challenge. The World report on road traffic accident prevention has indicated that worldwide, an estimated 1.2 million people die in road traffic accident each year and as many as 50 million are being injured [1]. Current and projected trends in motorization indicated that the problem of RTAs will get worse, leading to a global public health crisis. It has been indicated that, accordingly, by 2020 traffic accident is expected to be the third major killer after HIV/AIDS and TB [2].

Due to its perception as a 'disease of development', road traffic accidents and related injuries tend to be under-recognized as major health problems in developing countries. According to WHO report, 90% of the world's fatalities on the roads occur in low-income and middle-income countries, which have only 48% of the world's registered vehicles [1]. For example, an estimated total of 227, 835 pedestrians die in low-income countries, as opposed to 161,501 in middle-income countries and 22,500 in high income countries each year [3].

The severity of road traffic crashes is also likely to be much greater in Africa than anywhere else, because many vulnerable road users are involved, poor transport conditions such as lack of seat belts, overcrowding, and hazardous vehicle environments. The poor reporting system has also masked the magnitude of the problem in Africa. The lack of pre-hospital and hospital emergency care after accidents makes the outcome of car accidents in Africa the worst [4].

According to federal police commission report the death rate due to car accident is significantly increasing among pedestrians and passengers from time to time in Ethiopia [5]. A total of 25,110 accidents and 3,415 fatalities were recorded in Addis Ababa during 2000-2009. The majority of fatalities were pedestrian, 2970(87%) followed by passengers 297(9%) and drivers 148(4%) [6]. A report from traffic Police office of Mekelle town (the study area) indicated that in 2008, there were a total 313 RTAs and in 2009 the total number RTAs increased to 353. On the other hand, the report showed that 96% of the causes were related to human risk behavior whereas 4% was due to vehicle problem [7].

Evidences noted that human behavior is the most common factor accounting for more than 85% of all traffic accidents [2]. Among the risky human behaviors is driving over the recommended speed. Studies has indicated that an increase of 1 km/h in mean traffic speed results in a 3% increase in the incidence of accident crashes and a 4-5% increase in fatal crashes [8-10]. Another risky behavior identified for road traffic accident is taking alcohol and driving [11,12]. Not using seat belt while driving is additional risky behavior identified [13,14]. Mobile phoning while driving is becoming one of the riskier behaviors as well [15-17]. Knowledge, belief, attitude on risky driving behaviors and driving experience were also important aspects of risky behaviors identified with evidences [18-21]. Since evidences are directing us the most important factor for road traffic accident is human behavior, we have investigated the most important human factors of risky driving behavior for road traffic accident in Mekele city, northern part of Ethiopia.

## Methods

A cross--sectional quantitative study was conducted in Mekelle town, northern Ethiopia in April 2011. Mekelle is the capital city of Tigray national region state found in the northern part of Ethiopia. It is situated around 783 kms, north of Addis Ababa. According to 2007 Ethiopian central statistics report, the total population of Mekele city is 215, 540 (104,758 Male and 110,788 female) [22]. The total number of registered and licensed House cars, Taxi and Bajaj in 2011 were 931, 405 and 555 respectively [23]. We obtained the list of all the three kinds of vehicles in the area for sampling frame. The study was done among a sample of 350 drivers of Taxi, Bajaj (three tire vehicles) and private owned car drivers. After proportion to size allocation for Taxi (75), Baja (103) and private owned car (172) drivers, we used systematic random sampling method to identify illegible study subjects. Anyone who was driving a private owned car, Taxi and Bajaj in Mekelle town were included in the study. The study excluded drivers driving the targeted vehicles outside Mekelle and those who were sick and unavailable during the study period in Mekelle town.

To determine the sample size a single proportion population formula was employed. Taxi and Bajaj drivers identified with systematic random sampling were approached for the interview at their stations but the private owned cars were interviewed at their residential houses based on the information given from transport office of the town and taxi and Bajaj associations.

Trained personnel collected data by using pretested questionnaire adopted from literatures [24-26]. A questionnaire prepared in English was translated into the local language (Tigrigna) and back translated to check whether it is translated correctly or not. The instrument primarily measured road traffic accident as a collision between vehicles; between vehicles and pedestrians; between vehicles and animals; or between vehicles and fixed obstacle within 2 years period of time.

Risk behavior in this study is defined if a respondent has experienced any one of the four behaviors within the specified period. The behaviors are driving after drinking alcohol and/or, unfasten seat belt within the past 12 months; excessive speed which is above 35 Km/h within the past 6 months; mobile calling or receiving while driving within the past 12 months. The issue of seat belt is exempted for the drivers of Bajaj respondents since Bajaji is manufactured without belt.

The study posted 10 questions using photographs regarding drivers' knowledge about basic traffic signs on the roads implemented in the town and the country. These included photos of crossroad, Pedestrian crossing, students ahead, slow down your speed, automobiles no overtaking, closed to automobiles, no parking, no entry, no parking until you get another sign inscribed end, and maximum speed is 35 kms per hour and stop and give way. Respondents who answered below seven signs labeled as less knowledgeable, eight of the signs correctly as moderately knowledgeable and above eight signs correctly as high knowledgeable. Attitude about risky driving behaviors (driving with over speed, unfasten seat belt, mobile phone use while driving and drinking and driving) was measured using 16 items (four items for each behavior) and respondents were categorized as having unsupportive, indifferent and supportive attitude based on mean score of attitude measure.

After cleaning and editing the data was analyzed using SPSS version 16.0. Descriptive statistics was used to determine the frequency of dependent and independent variables. Variables which show significant association on biviarate analyses were fitted in to multivariate logistic regression model to determine the independent predicators for risky driving behavior. Ethical clearance was obtained from the Ethical committee of the college of Public Health and Medical Sciences, Jimma University. Consent was obtained from each respondent.

## Results

### Socio-demographic and background characteristics

Majority of the respondents 339 (96.9%) were males. The mean age of the study subjects was 28.7 (SD ± 9.9). More than half 202 (57.8%) were single in marital status. Majority 284(80.9%) of the study participants were Orthodox Christians. Majority of the respondents' educational status was secondary school 185 (52.8%) followed by primary school 122 (34.9%). Half of the respondents reported an average monthly family income below 2350 Eth Birr or approximately 167 USD (Table 1).

**Table 1:** Socio-Demographic Characteristics of Drivers of Mekelle Town, Northern Ethiopia, 2011

Variables	Frequency	Percent
**Sex**		
Male	339	96.9
Female	11	3.1
**Marital status**		
Single	202	57.8
Married	144	41.1
Other (Divorced, Widowed)	4	1.1
**Religion**		
Orthodox	283	80.9
Muslim	48	13.7
Catholic	11	3.1
Protestant	8	2.3
**Educational status**		
Primary school	122	34.9
Secondary	185	52.8
Tertiary level	43	12.3
**Average monthly income**		
Low	93	26.6
Middle	82	23.4
High	126	36.0
Very high	49	14.0

More than half of the respondents were driving their employers' vehicles 211 (60%). The mean driving year of experiences was 3.5 (SD ± 2.48). Within the period of the past 1 year, 66 (18.9%) of them had at least one experience of punishment or warning by traffic police for violating traffic rules and regulations. Half and quarter of the drivers 264 (75.4%) did not get any kind of advice or warning from important people they thought about risky driving behaviors. Among the 350 total respondents before the previous 2 years of the date of data collection, 77 (22%) of them ever had experience of accident while driving a vehicle.

### Knowledge on traffic signs and attitude on risky driving behaviors

Among the 10 traffic signs, five of them were answered correctly by the majority of the respondents i.e. 'four-way intersection is ahead' correctly answered by 347(99%), 'pedestrian is crossing' and 'no entry' by 349(99.7%) each, 'maximum speed is 35 kms per hour' by 307(87.7%) and 'stop and give way' answered correctly by 346(98.9%) respondents. About half 177(50.6%) only knew the sign 'no overtaking for automobile correctly' (Table 2). The overall knowledge score on the traffic signs indicated that 189 (54%), 61 (17.4%) and 100 (28.6%) of the respondents were high, moderate and less knowledgeable respectively. The finding has indicated that out of the 350 respondents 157 (44.9%), 12 (3.4%) and 181 (51.7%) had supportive/positive, neutral and unsupportive/negative attitude towards the risk driving behaviors respectively.

**Table 2:** Knowledge on traffic signs of Drivers in Mekelle Town, Northern Ethiopia, 2011

Items	Correctly identified No (%)	Not correctly identified No (%)
Four-way intersection is ahead.	347(99.0)	3(1.0)
Pedestrian is crossing, drive carefully!	349(99.7)	1(0.3)
Students ahead, slow down your speed!	271(77.4)	79(22.6)
No overtaking for automobile!	177(50.6)	173(49.4)
Closed to automobiles!	294(84.0)	56(16.0)
No parking!	210(60.0)	140(40.0)
No entry or crossing this way is not allowed!	349(99.7)	1(0.3)
No parking until you get another sign inscribed end!	287(82.0)	63(18.0)
Maximum speed is 35 kms per hour!	307(87.7)	43(12.3)
Stop and give way!	346(98.9)	4(1.1)

### Risky driving behaviors

From the total respondents, 148 (42.3%) of them were using mobile phone while driving vehicle The reasons given for why they used mobile phone were because of it was important for their business 43 (12.3%), it was difficult for them to ignore when calls came from friends and clients 104 (29.7%). On the other hand among the reasons given by non users were using mobile while driving would expose to accident 36 (10.3%), distract driving skill 20 (5.7%) and the other reason was in fear of 500 Eth birr punishment from traffic police 152 (43.4%).

Majority of the respondents 288 (82.3%) ever drink alcohol and 28 (9.7%) had an experience of drinking alcohol and driving within the last 12 months. The reasons given by the respondents for why they drove after drinking alcohol were; they believed that they were skillful and have self confidence in driving 23(6.6%) and to enjoy with friends after drinking 4 (1.1%).

Since Baja were manufactured without seat belt, all respondents of them 103(100%) were not using seat belt. Of the total of 247 respondents of house car and taxi, 97(62.6%) of house car and 58(37.4%) of taxi were unfasten their seat belt while driving. Some of the reasons given by the respondents of house car and taxi for not using seat belt were; seat belt did not have importance 35 (10.0%) and seat belt creates discomfort 119 (34.0%).

Significant proportion 303 (86.6%) of the respondents were following the recommended speed limit of driving in the town. The main reason for not following the speed limit by the respondents was someone who has skill full in driving did not see the importance of speed limit 99 (25.5%). In general, 233 (66.6%) of the drivers were found to be risk groups. Out of the total drivers with risky driving behavior, 129 (55.4%), 59 (25.3%) and 45 (19.3%) were house car, taxi and Bajaj drivers respectively.

### Determinants of risky driving behavior

Variables which had significant statistical association with risky driving behavior in bivariate analysis were entered into multivariate analysis. Based on the analysis, respondents with secondary/high school education had more chance [OR-2.5, 95%CI (1.02, 6.05)] of having risky driving behavior than people with university/college educational status. Compared to low income respondents, high [OR-8.5, 95%CI (3.03, 10.68)] and very high [OR-9.2, 95%CI (2.80, 11.46)] income respondents were at higher risk of driving behavior. Subjects who had supportive attitude towards risky driving behavior were [OR-13.7, 95%CI (3.31, 15.64)] times more likely to have risky driving behavior than respondents with unsupportive attitude. Drivers who did not get advice about risky driving behavior from significant others were more likely [OR-3.0, 95%CI (1.55, 5.78)] to have risky driving behavior compared with who had advice from significant others (Table 3).

**Table 3:** Logistic regression model showing predictors of risky driving behaviors of drivers in Mekelle town, North Ethiopia, 2011

Variable	Risky driving		AOR (95% CI)
		
	No Number (%)	Yes Number (%)	
**Educational status**			
Primary	45(36.9)	77 (63.1)	1.3 (0.46, 3.52)
Secondary	52 (28.1)	133 (71.9)	2.5 (1.02, 6.05)
Tertiary	20 (46.5)	23(53.5)	1.0
**Average monthly income**			
Low	45 (48.4)	48 (51.6)	1.0
Middle	30(36.6)	52 (63.4)	2.4 (0.96, 5.87)
High	32(25.4)	94 (74.6)	8.5 (3.03, 10.68)
Very high	10 (20.4)	39 (79.6)	9.2 (2.80, 11.46)
**Attitude towards risky driving behaviors**			
Supportive attitude	14 (8.9)	143 (91.1)	13.7 (3.31, 15.64)
Indifferent attitude	1(8.3)	11(91.7)	2.3 (2.26, 9.62)
Unsupportive attitude	102(56.4)	79 (43.6)	1.0
**Got advice from significant others**			
Yes	44 (51.2)	42 (48.8)	1.0
No	73 (27.7)	191 (72.3)	3.0 (1.55, 5.78)

## Discussion

This study showed that the proportion of subjects with risky driving behaviors was high. Higher educational status, having supportive attitude towards riskier driving behaviors and getting advice from significant others were predictors of risky driving behavior. On the other hand, high average monthly income was associated with risky driving behavior.

Though it is difficult to compare the difference of risky driving behavior between male and female drivers because of the limited number of female drivers, evidences have shown that males have more risky driving behavior than females due to biological and cultural reasons [27].

Studies have demonstrated that young drivers are frequently involved in highest proportion of risk behaviors and traffic accidents than other age groups [28,29]. Unlike the above studies, there was no significant difference among the different age groups in this particular study. This may be related with the age to get driving license in the area i.e. one needs to be more than 18 years old to get driving license by law.

Similar to a study done previously [17], our study also revealed that drivers with secondary or high school educational status had higher risky driving behaviors than drivers with university or college educational status. However, drivers with lower/primary educational status had no significant statistical difference in risky driving behavior with university or college educational status drivers. This may be related with drivers with lower educational status had more years of driving experiences as they got their driving license with old legislation; where, previously driving license was given even with lower educational status individuals in the area.

Increase in income was significantly associated with risky driving behavior in this study. Similar studies also identified that high income was associated with high risky driving behavior [17,30] This may be explained as average monthly income increases, the capacity to pay for vehicles and possible punishments or loses increases and drivers would be involved in risky driving behaviors.

Although the proportion of drivers without appropriate knowledge of traffic signs was not few; knowledge was not statistically significant predictor of risky driving behaviors. Rather unsupportive attitude about risky driving behaviors was highly associated with risky driving behaviors. In line to our study, other findings revealed that risky driving behaviors were found to be due to negative attitude rather than to poor knowledge about risky driving behaviors [18,31]. This evidence suggested to interventions against risky driving behavior to focus on attitude change rather than awareness and knowledge on risky driving behaviors.

A study noted that drivers who had more driving experience were found to exercise more risky behaviors [17]. In contrary, a study in Tanzania showed that drivers who were not having driving experience found to be with high risky driving behaviors [19]. Unlike to the above studies, our study showed that driving experience was not found as a predictor variable for risky driving behavior which needs further investigation for explanation. Similar to that of driving experience, having experience of car accident was not significantly associated with risky driving behavior. According to a study in Turkey, even if drivers encountered with car accidents their risk behavior may not be changed and most drivers perceive traffic accident as a result of fate [20]. This is also another area which needs further investigation whether such perception is similar in the study area or not.

Obtaining advice and/or warning from important others was positively associated with less risky driving behaviors. Similarly, a study done in Sweden reported that an attempt to persuade the young to intervene when they observe their friends (peer pressure) that are driving after alcohol consumption resulted in bringing positive achievements in risky driving behaviors [21]. This study is expected to suffer with recall and social desirability biases about the risky driving behaviors.

## Conclusions

Significant proportion of the study subjects were with high risky driving behaviors. Drivers of secondary education and with high average monthly income were more likely to have riskier driving behaviors. Having supportive attitude towards riskier driving behaviors increases the chance of developing risky driving behavior among drivers. Advice from significant others about risky driving behavior was one of the factor for reducing risky driving behavior. Interventions targeted at developing negative attitude towards riskier driving behaviors on drivers and significant others should be implemented to bring positive behavior change. The interventions need to be segmented with educational status and income.

## Competing interests

The authors declare that they have no competing interests.

## Authors' contributions

AH, AG and LA designed the study, analyzed the data and drafted the manuscript. EG was involved in the design, analysis of the data and drafting of the manuscript and critically reviewed the article. All authors read and approved the final manuscript.
